# Polycentric binding in complexes of trimethylamine-*N*-oxide with dihalogens†

**DOI:** 10.1039/d0ra08165e

**Published:** 2021-02-03

**Authors:** Olga M. Zarechnaya, Aleksei A. Anisimov, Eugenii Yu Belov, Nikolai I. Burakov, Alexander L. Kanibolotsky, Vasilii A. Mikhailov

**Affiliations:** L.M. Litvinenko Institute of Physical Organic and Coal Chemistry R. Luxemburg St., 70 Donetsk Ukraine v_mikhailov@yahoo.com; A.N. Nesmeyanov Institute of Organoelement Compounds, Russian Academy of Sciences 28 Vavilov St. 119991 Moscow Russia; D.I. Mendeleev Russian Chemical Technological University 9 Miusskaya Sq. 125047 Moscow Russia; WestCHEM, School of Chemistry, University of Glasgow Glasgow G12 8QQ UK

## Abstract

Dihalogens readily interact with trimethylamine-*N*-oxide under ambient conditions. Accordingly, herein, stable 1 : 1 adducts were obtained in the case of iodine chloride and iodine bromide. The crystal and molecular structure of the trimethylamine-*N*-oxide–iodine chloride adduct was solved. Furthermore, the geometry and electronic structure of the trimethylamine-*N*-oxide–dihalogen complexes were studied computationally. Only molecular ensembles were found in the global minimum for the 1 : 1 stoichiometry. The O⋯X–Y halogen bond is the main factor for the thermodynamic stability of these complexes. Arguments for electrostatic interactions as the driving force for this noncovalent interaction were discussed. Also, the equilibrium structures are additionally stabilised by weak C–H⋯X hydrogen bonds. Consequently, formally monodentate ligands are bound in a polycentric manner.

## Abstract 2

Dihalogens readily interact with trimethylamine-*N*-oxide under ambient conditions. Stable 1 : 1 adducts were obtained in a case of iodine chloride and iodine bromide. Formally monodentate ligands are bound in a polycentric manner.

## Graphical abstract

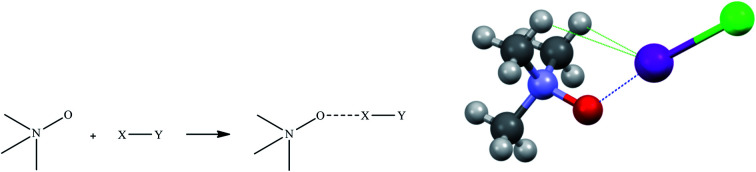

## Introduction

The relatively high reactivity of organic *N*-oxides^1^ and their compatibility with living organisms have been intriguing for a time. Recent studies have revealed not only the presence of trimethylamine-*N*-oxide (TMAO) in biota, including the human body,^2–6^ but also the huge variety of roles.^5–7^ The functional diversity of this substance makes it a promising target for pharmacological medicinal intervention.^2,8,9^ However, to date, only indirect methods have been used to regulate the TMAO level in mammalian tissues, such as dietary changes^5^ and probiotic/antibiotic control of the gut microbiota,^6^ both resulting in a slow response and low selectivity. Accordingly, more radical approaches may result in direct influence on the target by non-covalent interactions,^10^ mostly by hydrogen bonding. The proton affinity of trimethylamine-*N*-oxide^1,11^ in water (basicity) is not very high (compared with trimethylamine, for example), but its propensity to form hydrogen bonds is well known.^12^ For example, the hydrogen bonding controls the interaction of TMAO with urea.^13^ The pair “trimethylamine-*N*-oxide–urea” allows deep-water organisms to maintain osmotic resistance,^7^ avoiding protein denaturation at large carbamide concentrations. Other non-covalent TMAO interactions are less investigated, and only limited data for iodine complexation in dichloromethane has been presented.^14,15^ For new medicine development, another type of non-covalent interaction may be useful, namely halogen bonding.^16–18^ There is no data in the literature for halogen-bonded adducts of TMAO with an established structure, and even the possibility of TMAO binding with halogen bond donors is not discussed in numerous reviews^19–25^ devoted to halogen bonding, which is overlooked in prospects and conceptual articles.^26–32^ Diatomic molecules of halogens and interhalogens may be considered as the simplest donors of halogen bonds, and thus are convenient models for computation.

Dihalogens as donors pose a specific set of problems related to the possible coexistence of molecular complexes (where no breaking of covalent bonds occurs) and ionic complexes (formed due to heterolysis of a halogen–halogen bond).

An identical composition of these supramolecular aggregates makes it very difficult (or impossible) to discriminate ionic and molecular species by indirect structural methods. For uncharged nitrogen-centred nucleophiles, both molecular and ionic complexes are known, among them, the molecular complex pyridine–iodine^33^ and iodine cation coordinated with two pyridine molecules^34^ are most studied. For uncharged phosphorus-, sulphur- and selenium-centred nucleophiles, even more diverse patterns are found.^35,36^ For all known examples, halogen complex formation proceeds spontaneously, and to date, its direction cannot be controlled (entirely).

A few reports on computations for cationic halogen complexes^34^ demonstrated the thermodynamic stability of these forms. A number of computations for “halogen donor–heteroatom acceptor” interactions always led to molecular species (see reviews^22,23^ and references therein) with different degrees of covalent bond polarization. In the special case of exceptionally strong nucleophiles (carbenes^36^ and phosphines^37,38^), or ternary complexes,^39^ the covalent bond in a halogen donor lengthens up to breaking, then a new covalent bond “halogen-nucleophile” is formed, and a pair of ions mainly bound electrostatically appears. This may indicate the pathway to ionic complexes.

Although historically the first halogen bonded complexes were formed *via* the assistance of oxygen nucleophiles (chlorine clathrates,^40^ their structures as halogen bonded were revealed later^41^), it is not clear to date whether relatively low-nucleophilic oxygen species can stabilise cationic halogens. Among the uncharged oxygen nucleophiles, *N*-oxides are the strongest^11^ and the most probable candidates for the formation of ionic adducts.

Thus, to evaluate the possibility of a halogen bond between the oxygen centre of trimethylamine-*N*-oxide and halogens, we attempted to study the interaction of TMAO with molecular halogens and interhalogens by experimental and computational methods.

## Experimental

Acetonitrile (Labscane, Ireland, for synthesis) was purified to remove reductive impurities^42^ and stored over (preliminary activated at 400 °C) 3 Å molecular sieves.

Trimethylamine-*N*-oxide dihydrate (Acros Organics) was dehydrated by heating under reduced pressure (approx. 20–30 mm). Preliminary dehydrated TMAO was sublimed at a residual pressure less than 0.1 mm and obtained as snow-white needles, which rapidly deliquesced upon exposure to moist air. The sublimed compound was used for the synthesis of the complexes immediately.

### Synthesis of complexes of trimethylamine-*N*-oxide with halogens

#### Iodine chloride complex

Freshly sublimed trimethylamine-*N*-oxide (0.50 g; 6.66 × 10^−3^ mol) was dissolved in acetonitrile (5 mL), and a solution of iodine chloride (1.10 g; 6.77 × 10^−3^ mol) in acetonitrile was added under cooling and stirring. Approximately half of the solvent was removed from the reaction mixture under reduced pressure with gentle heating. The precipitated product was separated on a porous glass filter and washed with precooled acetonitrile (2 × 4 mL). Yellow crystals, mp 188–190 °C, yield 0.70 g (2.95 × 10^−3^ mol, 43%). Active halogen content was determined by iodometric titration, converting it to iodine chloride equals 68%. Analysis, %: C 15.30, H 3.66, I 52.50, Cl 14.70 (for the ratio I : Cl = 1 : 1). Calculated, %: C 15.17, H 3.82, I 53.44, Cl 14.93. Crystals for X-ray investigation were grown by the slow evaporation of the solution in acetonitrile.

The adduct of iodine bromide and trimethylamine-*N*-oxide (1 : 1 stoichiometry) was prepared using the same procedure as above, mp 171–173 °C. All attempts to isolate iodine complex gave products with a changeable content of active halogen, and the reasons for this are unclear. The complex with bromine was unstable at ambient temperature and rapidly converted into products of bromine reduction.

#### X-ray diffraction study

X-ray diffraction study of trimethylamine-*N*-oxide complex with iodine chloride was carried out using a SMART APEX2 CCD diffractometer (*λ*(Mo-Kα) = 0.71073 Å, graphite monochromator, *ω*-scans) at 100 K. Collected data was processed using the SAINT and SADABS programs incorporated in the APEX2 program package.^43^ The structures were solved by direct methods and refined by the full-matrix least-squares procedure against *F*^2^ in anisotropic approximation. Positions of hydrogen atoms were located from the Fourier difference map and refined isotropically without restrains. The refinement was carried out with the SHELXTL program.^44^

Crystallographic data for the trimethylamine-*N*-oxide–iodine chloride adduct: C_3_H_9_NO·ICl are orthorhombic, space group *Pnma*: *a* = 9.60490(10) Å, *b* = 7.63670(10) Å, *c* = 9.63370(10) Å, *V* = 706.629(14) Å^3^, *Z* = 4, *M* = 237.46, *d*_cryst_ = 2.232 g cm^−3^. w*R*^2^ = 0.0343 calculated on *F*_*hkl*_^2^ for all 1187 independent reflections with 2*θ* < 62.0°, (GOF = 1.281, *R* = 0.0140 calculated on *F*_*hkl*_ for 1182 reflections with *I* > *2σ*(*I*)). Crystallographic data (excluding structure factors) for the structure has been deposited at the Cambridge Crystallographic Data Centre (CCDC) as supplementary publication no. CCDC 2004829.†

### Computational details

The geometries, total electron energies, and wave functions were calculated using ORCA^45^ in the frame of density functional theory (DFT) with full optimization, without any limitations on symmetry. The DGDZVP all-electron split-valence basis with polarization functions was used for all atoms, in combination with the B3LYP hybrid functional. This basis set is optimized for DFT calculations of compounds with heavy atoms.^46,47^ The combination B3LYP/DGDZVP is an inexpensive and effective method for the study of halogen compounds.^48,49^ This makes possible to get reasonable estimations of energy, geometry and electron distribution without the use of pseudopotentials for heavy halogens, thus avoiding uncertainties during topological analysis^50^ of electron density distribution in terms of the “atom-in-molecules” theory.^51^ The presence of true minima was confirmed by the absence of imaginary frequencies in the harmonic vibrational mode calculations.

Energy of complexation, Δ*E*, was calculated as the difference between the total energy of a complex and the sum of energies of non-bound acceptor and donor molecules under their equilibrium geometry:

1 Δ*E* = *E*_complex_ − ∑(*E*_acceptor_ + *E*_donor_)

Total energy, *E*, was corrected to standard conditions (298.15 K, 1 atm) using zero-point vibrational energies (ZPVE) and corrections for enthalpy and free energy. These values were used for evaluation of the thermochemical characteristics of complex formation (Gibbs energy, Δ*G*, and enthalpy, Δ*H*) according to eqn (1). In the particular case of the TMAO⋯I–I complex, the values of *E*, Δ*E*, Δ*G* and Δ*H* were calculated for the virtual medium dichloromethane under continuum approximation in the form of SMD.^52^ Geometry and electron wave functions were also calculated for the TMAO⋯I–Cl complex for virtual acetonitrile under SMD^52^ and CPCM.^53^

Complexation energies were also calculated with corrections for basis set superposition errors (*E*_BSSE_) by the method of Boys and Bernardi^54^ with geometry relaxation. The BSSE-corrected thermodynamic characteristics were evaluated by

2 Δ*G*_BSSE_ = Δ*G* + *E*_BSSE_

3 Δ*H*_BSSE_ = Δ*H* + *E*_BSSE_

The electron density distribution was analysed with Multiwfn v.3.7.^55^ The electrostatic potential distribution was calculated^56^ for the 0.001 a.u. isodensity surface. The electron density distribution and electrostatic potential were visualized in VMD v.1.9.3,^57^ and all diagrams were built in SciDAVis.^58^ Hirshfeld surfaces were generated with CrystalExplorer.^59^

## Results and discussion

**Scheme 1:**
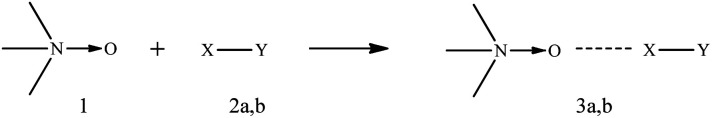
Formation of molecular complexes upon the interaction of dihalogens with trimethylamine-*N*-oxide (1). X = I, Y = Cl for 2a and 3a; X = I, Y = Br for 2b and 3b.

The interaction of trimethylamine-*N*-oxide with iodine chloride and iodine bromide leads to the formation of adducts with a 1 : 1 stoichiometry (Scheme 1).

The oxygen coordination to iodine in iodine chloride adduct 3a was revealed by X-ray investigation, as was expected from previous experience. Most probably, in iodine bromide adduct 3b, the same coordination occurred. To avoid any confusion, all virtual (computed) structures of the trimethylamine-*N*-oxide–dihalogen complexes will be denoted as TMAO⋯X–Y (TMAO⋯ICl represents the calculated structure of the virtual analogue of the experimentally isolated adduct 3a).

### Geometry of starting ligands and complexes

#### Trimethylamine-*N*-oxide

The calculations in B3LYP/DGDZVP reproduced the geometry of the trimethylamine-*N*-oxide free molecule well (Table 1), where the differences between the experiment and calculations in bond lengths do not exceed 0.007 Å, and the discrepancies in angles are less than one degree.

**Table 1:** Experimental and calculated bond lengths and angles in the molecule of trimethylamine-*N*-oxide

	*r*_N–O_, Å	*r*_N–C_, Å	∠CNO, degrees	∠CNC, degrees
B3LYP/DGDZVP (this paper)	1.372	1.503	109.5	109.5
Gas phase electron diffraction^60^	1.379	1.496	108.9	110.0
X-ray in crystal^61^	1.388	1.479	109.9	109.0
Computational HF/6-31G* (ref. 60)	1.370	1.473	108.7	110.2
Computational B3LYP/6-311G* (ref. 62)	1.367	1.501	109.6	109.3
Computational MP2/6-311+G* (ref. 62)	1.361	1.489	109	109.4
Computational PBE0/(aug-cc-pVTZ)^63^	1.348	1.487		

#### Halogen and interhalogens

The calculated interatomic distances for the diatomic halogens differ from the experimental values by no more than 0.07 Å (Table 2). In general, this exceeds the error of routine structural experiments. Interhalogen bonds are susceptible to pressure and temperature,^64^ and influence from the neighbouring atoms. Consequently, even larger basis set calculations do not always provide better agreement with the experiment.^65,66^

**Table 2:** Calculated and experimentally measured bond lengths (in Å) for diatomic halogens^a^

Halogen	F–F	Cl–Cl	Cl–F	Br–Br	Br–F	Br–Cl	I–I	I–F	I–Cl	I–Br
B3LYP/DGDZVP (this paper)	1.411	2.057	1.671	2.341	1.811	2.200	2.744	1.983	2.399	2.538
Experimental X-ray or neutron diffraction in crystal or liquid	*α*: 1404 (ref. 67)	1.991;^68^ 1.985;^69^ 1.994 (ref. 64)	1.628 (ref. 69)	2.301 (ref. 64)	1.822 (ref. 70) in complex with CH_3_Cl	2.178 (ref. 70)	2.715;^71^ 2.710 (ref. 65)		*α*: 2.37, 2.44;^72^*β*: 2.35, 2.44 (ref. 73)	2.52 (ref. 74)
Experimental spectroscopy in gas	1.4119 (ref. 75)	1.9879 (ref. 75)	1.628 (ref. 75)	2.281 (ref. 75)	1.759 (ref. 76)	2.138 (ref. 77)	2.666 (ref. 75)	1.9097 (ref. 75)	2.321 (ref. 77)	2.469 (ref. 75)
Computational	1.415;^67^ 1.4181 (ref. 79)	2.0195;^79^ 2.028 (ref. 81)	1.6464 (ref. 79)	2.279 (ref. 66)	1.736 (ref. 70)	2.1435 (ref. 78)	2.798;^65^ 2.654 (ref. 66)	1.9110 (ref. 78)	2.313;^66^ 2.513 (ref. 80)	2.462;^66^ 2.677 (ref. 80)

a Ccomputations were made in B3LYP-D/DZV,^65^ PBE0/def2-TZVPPD,^66^ CCSD(T) aug-cc-pVTZ/,^67^ MP2/aug-cc-pVTZ,^70^ CCSD(T)/aug-cc-pV(5+d)Z and CCSD(T)/aug-cc-pRV5Z,^78^ CCSD(T)/aug-cc-pVTZ,^79^ B3LYP/LANL2DZ,^80^ and MP2/6-311++G(2D)^81^ (for dimer). For a large set of precise computations for dihalogens, see ref. 78.

#### The electron density distribution

The electron density distribution in the molecules of the starting ligands and complexes was interpreted in terms of Bader's concept “atoms in molecules” (quantum theory of atoms in molecules, QTAIM).^51^ In the frame of this concept, every atom is defined by its own basin. The boundaries of the basins are determined by zero flux of electron density, ∇*ρ*(*r*) ⋅ *n⃑* = 0. The electron population of this basin is characterized by the integral of electron density upon the whole basin volume plus the nucleus charge, and value *Ω* is usually called the “net charge” or “Bader charge”, analogously to the “Mulliken charge”. The bond critical point and bond path are necessary and sufficient conditions for bonding due to QTAIM.^82^ The suitability of these metrics has been considered questionable many times (see, for example ref. 83), but paradigmatic discussions are beyond the scope of this paper. Herein, the “net charge” *Ω* is given in fraction of elementary charge (1*e* = 1.602 × 10^−19^ C) and electron density *ρ*(*r*) in the bond critical points (bcp) is given in atomic units (a.u., e bohr^−3^; 1 bohr = *a*_0_ = 0.529 Å). In accordance, Laplacian ∇^2^*ρ*(*r*) is given in e bohr^−5^, density of potential energy *V*(*r*) and kinetic energy *G*(*r*) in hartree bohr^−3^. These values are presented in Table 3, with a limited number of literature experimental^65,81^ and computational^66^ results.

**Table 3:** Topology of electron distribution for free molecules of halogen and interhalogens

Halogen/interhalogen X–Y	*ρ*(*r*), e bohr^−3^	∇^2^*ρ*(*r*), e bohr^−5^	*G*(*r*), hartree bohr^−3^	*V*(*r*), hartree bohr^−3^	*Ω*_X_, e
F–F	0.272	0.552	0.273	−0.409	0
Cl–Cl	0.128; 0.149 (ref. 81)	0.050	0.057	−0.101	0
Cl–F	0.191	−0.074	0.129	−0.278	0.378
Br–Br	0.095	0.016	0.036	−0.068	0
Br–Cl	0.108	0.031	0.046	−0.084	0.109
Br–F	0.139	0.298	0.1379	−0.201	0.461
I–I	0.064; 0.050;^65^ 0.079 (ref. 66)	0.015; 0.082 (ref. 65)	0.0214	−0.039	0
I–Br	0.0760; 0.094 (ref. 66)	0.0340	0.0310	−0.054	0.176; 0.187 (ref. 66)
I–Cl	0.0826; 0.10 (ref. 66)	0.0710	0.042	−0.067	0.266; 0.323 (ref. 66)
I–F	0.106	0.340	0.1205	−0.156	0.567

Table 4 presents some data for the electron distribution in the TMAO molecule around the heavy atoms and the oxygen–nitrogen bond critical point. Only computational results are available for comparison (Table 4). In one case, only the sum ∑*d*(*r*) = *G*(*r*) + *V*(*r*) was given in the literature.^62^

**Table 4:** Topology of electron distribution (N–O bond) for free molecule of trimethylamine-*N*-oxide

Method	*ρ*(*r*), e bohr^−3^	∇^2^*ρ*(*r*), e bohr^−5^	*G*(*r*), hartree bohr^−3^	*V*(*r*), hartree bohr^−3^	*E*(*r*), hartree bohr^−3^	*μ*, D	*Ω*_O_, e	*Ω*_N_, e
B3LYP/DGDZVP (this paper)	0.335	−0.124	0.231	−0.500	−0.269	4.85	−0.701	−0.43
B3LYP/6-311+G* (ref. 62)	0.349	−0.293	—	—	−0.305	5.05	−0.70	−0.43
MP2/6-311+G* (ref. 62)	0.353	−0.326	—	—	−0.332	5.26	−0.74	−0.48
PBE0/aug-cc-pVQZ^63^	0.367	−0.28	0.21	−0.48	−0.27	—	−0.74	−0.43

As can be seen from Tables 1–4, the computations in B3LYP/DGDZVP reproduced the experimental geometry of TMAO and halogens molecules adequately and produced reasonable estimations for its electron density distribution. This degree of conformity gives hope to find credible structures for complexes of TMAO and halogens.

#### TMAO–halogen complexes

**Fig. 1:**
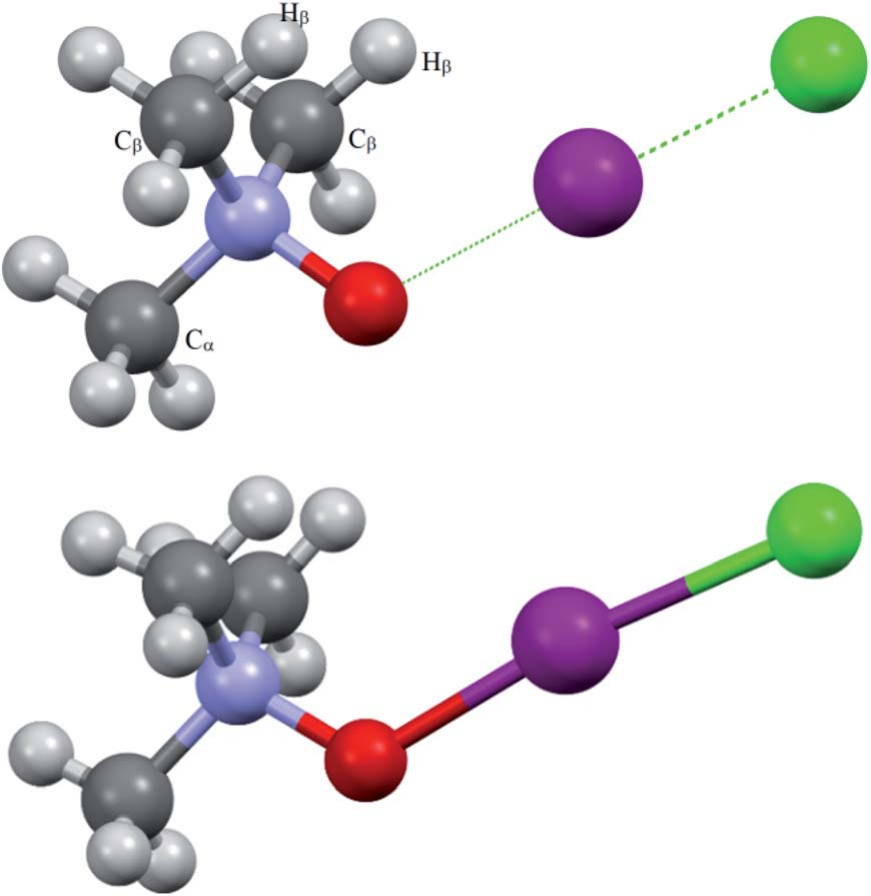
Computed (top) and experimentally solved (bottom) structures of the TMAO⋯ICl complex and adduct 3a.

The overall view of the virtual complex TMAO⋯ICl (computed structure) and adduct 3a (experimentally solved structure) is presented in Fig. 1.

**Fig. 2:**
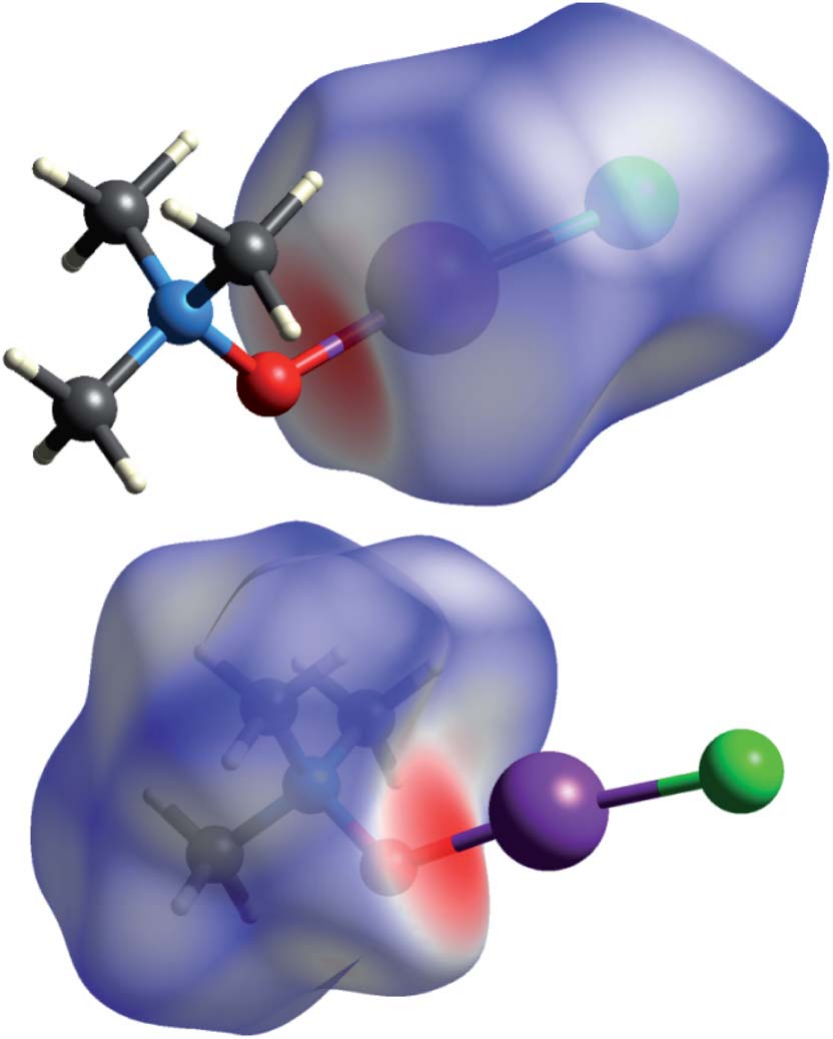
Hirshfeld surfaces for ICl (top) and TMAO (bottom) fragments of adduct 3a.

Short contacts O⋯I and H⋯I are clearly visible on the Hirschfeld surfaces generated separately for the *N*-oxide and iodine chloride fragments of adduct 3a (Fig. 2).

Some geometry parameters of the computed structures of the trimethylamine-*N*-oxide–halogen complexes are presented in Table 5.

**Table 5:** Interatomic distances (Å) and angles (degrees) in the computed structures of trimethylamine-*N*-oxide–halogens complexes

	Complex	*r*_X–X_, Å	*r*_X–O_, Å	*r*_N–O_, Å	*r*_N–C_, Å	*r*_X–H_, Å	∠X–X–O, degrees	∠X–O–N, degrees	∠∠X–O–N–C_α_, degrees	∠∠X–O–N–C_β_, degrees	∠∠X–O–C_β_–H_β_, degrees
1	TMAO⋯F–F (B3LYP)	1.636	1.916	1.399	1.499(*α*); 1.505(*β*)	2.267; 2.268	175.41	100.62	179.89	61.14; 60.93	3.40; 3.85
2	TMAO⋯Cl–Cl (B3LYP)	2.205	2.271	1.398	1.500(*α*); 1.503(*β*)	2.790, 2.792	179.17	113.85	179.97	61.16, 61.08	3.35; 3.46
3	TMAO⋯Cl–F (B3LYP)	1.783	2.173	1.402	1.499(*α*); 1.503(*β*)	2.773; 2.765	178.80	114.30	179.49	61.68; 60.69	3.25; 4.43
4	TMAO⋯Br–Br (B3LYP)	2.453	2.368	1.399	1.500(*α*); 1.503(*β*)	2.916, 2.916	178.86	116.31	180.0	61.11, 61.11	4.75, 4.75
5	TMAO⋯Br–Cl (B3LYP)	2.323	2.321	1.401	1.500(*α*); 1.503(*β*)	2.900, 2.900	178.77	116.34	180.00	61.18, 61.19	4.83, 4.83
6	TMAO⋯Br–F (B3LYP)	1.906	2.249	1.404	1.499(*α*); 1.503(*β*)	2.867	178.15	116.36	179.90	61.14; 61.32	4.86; 5.10
7	TMAO⋯I–I (B3LYP)	2.844	2.534	1.398	1.500(*α*); 1.503(*β*)	3.139, 3.139	178.44	120.44	180.0	61.08, 61.09	7.10, 7.10
8	TMAO⋯I–Br (B3LYP)	2.641	2.481	1.401	1.500(*α*); 1.503(*β*)	3.108, 3.108	178.47	120.23	180.00	61.17, 61.17	7.03, 7.03
9	TMAO⋯I–F (B3LYP)	2.055	2.403	1.404	1.499(*α*); 1.503(*β*)	3.078; 3.051	177.81	119.87	178.20	63.04; 59.46	5.07; 8.91
10	TMAO⋯I–Cl (B3LYP)	2.506	2.444	1.402	1.500(*α*); 1.503(*β*)	3.087, 3.087	178.61	120.09	180.00	61.22, 61.22	6.95, 6.94
11	TMAO⋯I–Cl (B3LYP/D3)	2.505	2.470	1.401	1.500(*α*); 1.503(*β*)	3.072; 3.072	179.28	119.08	180.00	61.12; 61.12	7.03; 7.03
12	TMAO⋯I–Cl (CAM-B3LYP/D3)	2.469	2.447	1.390	1.489(*α*); 1.492(*β*)	3.050; 3.112	178.12	120.41	176.78	57.84; 64.22	10.65; 4.82
13	TMAO⋯I–Cl (M06-2X)	2.452	2.420	1.387	1.490(*α*); 1.492(*β*)	3.029; 3.043	179.41	119.38	179.09	61.97; 60.16	6.63; 8.52
14	TMAO⋯I–Cl B3LYP/CH3CN/CPCM	2.610	2.298	1.421	1.504(*α*); 1.502(*β*)	3.096	175.43	121.10	180.00	64.35; 64.36	8.80; 8.81
15	TMAO⋯I–Cl B3LYP/CH3CN/SMD	2.633	2.274	1.425	1.501(*α*); 1.499(*β*)	3.089	174.67	120.80	179.87	61.92; 61.67	8.45; 8.81

X-ray analysis of the single crystal of adduct 3a revealed the following geometry parameters (lengths in angstroms, angles in degrees): *r*_I–Cl_ 2.5685(5), *r*_I–O_ 2.1895(15), *r*_N–O_ 1.422(2), *r*_N–C_ 1.491(3) (*α*), *r*_N–C_ 1.4943(18) (*β*), *r*_I–H_ 3.071(21), ∠Cl–I–O 172.93(4), ∠I–O–N 119.89(11), ∠∠I–O–N–C_α_ 180.000(0), and ∠∠I–O–N–C_β_ 61.61(11). The general appearance of all the computed structures is very similar, and the structure of the iodine chloride complex is shown in Fig. 1. The I–Cl and N–O covalent bonds are longer in the real structure 3a (lengthened and weakened to a greater extent) compared to the computed TMAO⋯ICl (entry 10), and contact O⋯I is significantly shorter. Other functionals with embedded dispersion corrections (entries 12 and 13) did not change this tendency. One of the possible reasons is the influence of the crystal field in the solid phase, as was shown for complexes of *N*-haloimides with pyridines.^84^ An attempt was made to model this influence with media besides vacuum. Acetonitrile was chosen as a highly polar aprotic solvent, as represented by two widely used polarized continuum models, namely CPCM (entry 14) and SMD (entry 15). Both models gave similar results, where contact O⋯I became shorter and closer to that found in experimental structure 3a, and the covalent I–Cl bond became even longer compared with the crystal structure. In the crystal, all the components of the complex are tightly surrounded with neighbours (Fig. 2), and thus the polarized continuum is not sufficient to account for these interactions. It is worth mentioning that both models still predicted the molecular organization of the complex (not ionic).

All the computed structures of TMAO⋯X–Y have some common features, including two halogen atoms and an oxygen atom lying in the same straight line; the distance between the oxygen and halogen atoms is less than the sum of the van der Waals radii; the halogen–halogen covalent bond in the complex is longer than in the starting halogen molecule; and the nitrogen–oxygen covalent bond in the complex is longer than in the starting *N*-oxide.

These features are common for all known halogen complexes with uncharged heteroatom nucleophiles, as was noted in early studies.^85^ The specific features of trimethylamine-*N*-oxide complexes are the sharp difference between their methyl groups (initially equivalent) and two short contacts C–H⋯Hal. There are two types of methyl groups in the complex structure as follows: (a) the α-methyl group is anti-periplanar to the nearest halogen atom in relation to the N–O bond and (b) two symmetrical β-methyl groups are in the gauche conformation relative to the proximal halogen (are synclinal to this atom relative to the N–O bond). The dihedral angle (α-C)–N–O–Hal is very close to a straight angle for all the computed structures and the torsions (β-C)–N–O–Hal vary around 60°. In this orientation, one C–H bond of every β-methyl group becomes almost parallel to the oxygen–halogen bond with a low dihedral angle value for Hal–O–C_β_–H_β_ (see Table 5).

This arrangement of methyl groups and halogen is favourable for short contact and C_β_–H_β_⋯Hal hydrogen bond formation; however, it precludes any type of interaction between the α-methyl group and halogen atom in the computed structures. Consequently, the interatomic distance of C_β_–H_β_⋯Hal is less than the sum of the van der Waals radii for the hydrogen and halogen atoms (Table 5). This type of contact shortening is well known for the subtle C–H⋯X (X = halogen) hydrogen bond.^23,86–88^

The heteroatom X⋯O contacts in the complexes under study may be compared with the shortest known contacts in the experimental structures, including 4,4′-dipyridyl-*N*,*N*′-dioxide–1,4-diiodotetrafluoro-benzene of 2.725 Å;^89^ 4-methylpyridine-*N*-oxide–*N*-iodosaccharine of 2.276 Å and 4-methoxypyridine-*N*-oxide–*N*-iodosaccharine of 2.295 Å;^90^ and 4-dimethylaminopyridine-*N*-oxide–iodine^91^ of 2.359 Å. It is clear that the I⋯O contact in adduct 3a is the shortest between the experimentally studied structures. Thus, it is reasonable to suppose that the interaction between trimethylamine-*N*-oxide and iodine chloride is the strongest for all the studied halogens and *N*-oxides (and more generally, between all the studied halogens and uncharged oxygen nucleophiles).

### Energy for TMAO–halogen complex formation

The energy values for the formation of the TMAO–halogen complex are presented in Table 6. The zero point energy corrections turn out to be sufficient for the studied structures (and BSSE corrections). The influence of the basis set superposition has been acknowledged for complexes of iodine^92^ and iodine halides,^80^ and for structures in which the halogen atom is involved in halogen and hydrogen bonding.^93^ In this case, this may not be a property of these atoms or complexes, rather the basis sets are not large enough. Superposition corrections are significantly lower under augmented basis sets computations.^79^ Thus, the geometry of the complexes and electron density topology were not sensitive to the basis set superposition, and corrections are negligible.

**Table 6:** Interaction energies for TMAO⋯X–Y

Complex	Δ*E*, kJ mol^−1^	Δ*E*, ZPVE corr., kJ mol^−1^	Δ*E*, BSSE corr., kJ mol^−1^	Δ*G*, BSSE corr., kJ mol^−1^
TMAO⋯F–F	−61.20	−55.32	−118.91	−13.12
TMAO⋯Cl–Cl	−56.89	−51.73	−69.45	−9.52
TMAO⋯Cl–F	−82.76	−76.39	−94.39	−30.05
TMAO⋯Br–Br	−65.51	60.58	−69.87	−15.97
TMAO⋯Br–Cl	−76.39	−71.16	−83.26	−24.14
TMAO⋯Br–F	−103.61	−97.50	−108.37	−47.73
TMAO⋯I–I	−64.85	−60.53	−64.31	−14.92
TMAO⋯I–Br	−78.03	−73.36	−78.62	−24.93
TMAO⋯I–Cl	−88.63	−83.63	−89.75	−34.03
TMAO⋯I–F	−106.30	−100.38	−105.98	−49.08

Thermodynamic data for the formation of a complex of diiodine with trimethylamine-*N*-oxide in dichloromethane allowed the direct comparison of the experimental Gibbs energy^14,15^ with the calculated energy of complexation, as estimated in this paper. The reported Δ*G* values for this 1 : 1 equilibrium are −18.4 kJ mol^−1^ (ref. 15) and −21.4 kJ mol^−1^ (calculated from the data in ref. 14; original text indicates Δ*H* = −10 kcal mol^−1^ and ΔS = −16.9 e.u.). The maximum difference (6.5 kJ mol^−1^ in comparison with data in ref. 14) seems to be a significant disagreement. Computations accounting for the influence of the medium (virtual dichloromethane, *ε* = 8.93, instead of vacuum; see Experimental section and computational details) gave a Δ*G* value (−20.98 kJ mol^−1^) more negative compared to that for vacuum (−14.92 kJ mol^−1^), and closer to the aforementioned experimental values.^14,15^ Comparable values for the interaction energies were calculated for 4-dimethylaminopyridine-*N*-oxide⋯I–I (−12.948 kcal mol^−1^ (ref. 91)) and pyridine-*N*-oxide⋯*N*-iodosaccharine (−70.2 kJ mol^−1^ (ref. 90)). In general, the calculated interaction energies, Δ*E*, resemble the corresponding values for the strongest hydrogen-bonded complexes^94^ and halogen-bonded complexes.^80,95,96^ For these systems, the BSSE corrections were also found to be sufficient. Computations in MP2 usually give slightly lower values for interaction energies in comparison with DFT/B3LYP.^80,95^

### Electrostatic potential distribution in ligands and complexes

The structural motifs of the complexes under study became clearer after considering the electrostatic potential (ESP) on the surface of *N*-oxide and the halogen molecules. The surface potential was calculated for the 0.001 a.u. boundary density, which was done in the majority of papers referenced in Table 7.^97–106^ In one paper,^96^ calculations were performed for the 0.002 a.u. boundary.

**Table 7:** Maximum values of positive surface electrostatic potential *V*_S,max_ (magnitude of σ-hole) for halogens and interhalogens

Halogen (interhalogen)	*V*_S,max_, kcal mol^−1^	Literature data
F–F	14.67	13.8;^97^ 12.95 (ref. 98)
Cl2	24.85	27.60;^98^ 23.8;^99^ 25.1;^100^ 28.2;^101^ 28.6305;^102^ 24.8 (ref. 103)
Cl–F	45.61	40.79;^39^ 45.8;^101^ 45.0 (ref. 108)
Br2	31.60	32.0;^96^ 29.1;^99^ 31.8;^101^ 32.0;^102^ 27.9 (ref. 103)
BrCl	38.78	37.89;^96^ 37.8;^101^ 37.9 (ref. 102)
Br–F	56.14	56.72;^96^ 56.3;^101^ 47.5;^104^ 58.35843;^105^ 53.0 (ref. 108)
I2	32.92	48.10;^96^ 30.25;^103^ 40.45 (ref. 106)
IBr	40.58	60.91 (ref. 96)
ICl	47.57	72.35 (ref. 96)
I–F	59.41	91.49 (ref. 96)

**Fig. 3:**
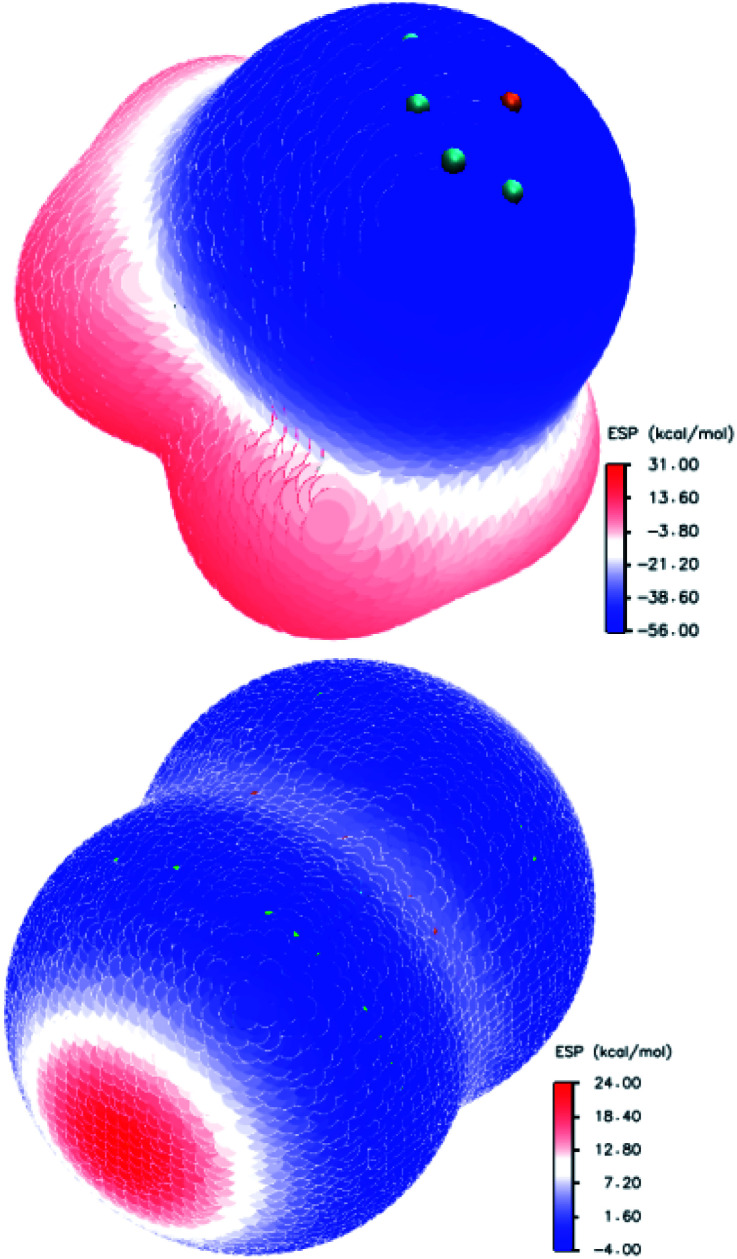
Electrostatic potential distribution on the molecular surface of trimethylamine-*N*-oxide (top) and chlorine (bottom).

The oxygen atom in the molecule of trimethylamine-*N*-oxide forms a hemispheric surface with a negative potential (Fig. 3, left). Localisation of the most negative potential (−55.97 kcal mol^−1^) may be described as a circumference on this sphere (green dots, Fig. 3) with a centre on a crossover point of continuation of the N–O bond and boundary surface with an electron density of 0.001 a.u. At this crossover point (orange point), the potential is still negative (−55.64 kcal mol^−1^), but this is the local minimum in comparison with vicinal areas. The distribution of the ESP on the surface of the chlorine molecule (Fig. 3, right side) is typical for diatomic halogens and interhalogens, where the areas with the most positive potentials are localised on the outermost region of the halogen surface centred on the “halogen–halogen” axis, with toroidal areas of negative potential encircling this axis. This type of ESP distribution in halogens is well established and discussed many times in the literature.^107,108^ Imaginably, the interaction of the positively charged area (σ-hole) of halogens with the negatively charged area of *N*-oxide leads to structures with a short N⋯O contact.

The quantitative assessment of the ESP depends on the method used, and some literature data for halogens is presented in Table 7 for comparison. Different units were used by different authors (1 hartree = 4.3597 × 10^−18^ J = 627.51 kcal mol^−1^ = 2625.5 kJ mol^−1^ = 27.211 eV), and kcal mol^−1^ was used most widely; also, the literature data from ref. 39, 102, 103 and 105 was recalculated. The largest discrepancies are obvious for iodine compounds. Possibly, this is a result of the pseudopotential used in the computations for these compounds^96,106^ and the different envelopes (0.002 a.u. for data from ref. 96). The divergence between the B3LYP/DGDZVP results (current paper) and QCISD^103^ or CAM-B3LYP/TZVPD^103^ (Table 7) is much less pronounced. Expectedly, the positive potential *V*_S,max_ increases with atom X (heavy atom in interhalogens) in the order of F < Cl < Br < I. For the Y atom in the interhalogens, the order of influence on the X σ-hole is the opposite.

### Electron distribution in trimethylamine-*N*-oxide complexes with halogens

Complex formation with trimethylamine-*N*-oxide leads to bond polarization in homoatomic halogen molecules (chlorine, bromine, and iodine) and reinforces polarization in interhalogen molecules, where the electron density in the halogen atom basins changes significantly (Table 8). The majority of X halogens connected to oxygen lose their electron density compared with the starting molecule. In contrast, the fluorine X basin is enriched in the complex with TMAO, and the *Ω* value becomes markedly negative. In this virtual structure, two heteroatoms, oxygen and fluorine, with electronically enriched basins (negative *Ω*) interact closely. This is not unique for fluorine in contact with oxygen.^109^ The value Δ*q* (fraction of elementary charge) in the last column of Table 8 corresponds to the overall charge transfer from the *N*-oxide molecule to a halogen molecule. Some counterintuitive changes occur in the trimethylamine-*N*-oxide fragment. The molecule of *N*-oxide as a whole loses electron density. The population of carbon atoms does not change significantly. The electron populations of hydrogen and oxygen basins diminish, but the electron density in the nitrogen atom basin increases for all the complexes. It is worth mentioning that in the experimentally studied complexes of *N*-oxides with the *N*–O⋯I halogen bond,^110^ the population of nitrogen basins also increases compared to free *N*-oxide. The overall charge transfer, Δ*q*, depends mainly on the nature of X atom (closest to oxygen). For TMAO⋯I–I, this value is comparable with the charge transfer in complexes of diiodine with pyridine-*N*-oxides.^91^

**Table 8:** Net charge, *Ω* (fraction of elementary charge), of heavy atoms and charge transfer in trimethylamine-*N*-oxide complexes with halogens

Complex	*Ω*_O_, e	*Ω*_N_, e	*Ω*_X_, e	*Ω*_Y_, e	Δ*q*, e
TMAO⋯F–F	−0.4371	−0.490	−0.132	−0.286	−0.42
TMAO⋯Cl–Cl	−0.582	−0.485	−0.00292	−0.234	−0.24
TMAO⋯Cl–F	−0.592	−0.491	0.263	−0.495	−0.23
TMAO⋯Br–Br	−0.614	−0.485	0.0291	−0.242	−0.21
TMAO⋯Br–Cl	−0.613	−0.488	0.124	−0.326	−0.20
TMAO⋯Br–F	−0.617	−0.494	0.371	−0.574	−0.20
TMAO⋯I–I	−0.659	−0.481	0.0960	−0.239	−0.14
TMAO⋯I–Br	−0.659	−0.486	+0.266	−0.377	−0.11
TMAO⋯I–Cl	−0.662	−0.491	+0.262	−0.445	−0.18
TMAO⋯I–F	−0.668	−0.492	+0.510	−0.659	−0.15

### Electron density in the bond critical points

The bond critical points (3,−1) for the short contact O⋯Hal were revealed for all the TMAO⋯X–Y complexes under study, and for these BCPs, the electron density *ρ*(*r*), Laplacian of electron density ∇^2^*ρ*(*r*), potential energy density *V*(*r*) and kinetic energy density *G*(*r*) were calculated (Table 9). A low density BCP, positive (plus sign) Laplacian, and close to unit ratio of potential energy density *V*(r) to kinetic energy density *G*(*r*) (last column in the Table 9) are typical for closed shell interactions.^111^

**Table 9:** Some characteristics of the electron density in O⋯Hal BCPs for trimethylamine-*N*-oxide complexes with halogens

Complex	*ρ*(*r*), e bohr^−3^	∇^2^*ρ*(*r*), e bohr^−5^	*G*(*r*), hartree bohr^−3^	*V*(*r*), hartree bohr^−3^	|*V*|/*G*
TMAO⋯F–F	0.0755	0.297	0.0770	−0.0797	1.04
TMAO⋯Cl–Cl	0.0514	0.157	0.0405	−0.0417	1.03
TMAO⋯Cl–F	0.0624	0.173	0.0484	−0.0534	1.10
TMAO⋯Br–Br	0.0470	0.130	0.0346	−0.0366	1.06
TMAO⋯Br–Cl	0.0512	0.137	0.0378	−0.0413	1.09
TMAO⋯Br–F	0.0594	0.147	0.0447	−0.0526	1.18
TMAO⋯I–I	0.0390	0.106	0.0278	−0.0291	1.05
TMAO⋯I–Br	0.0428	0.116	0.0314	−0.0338	1.08
TMAO⋯I–Cl	0.0451	0.123	0.0339	−0.0370	1.09
TMAO⋯I–F	0.0489	0.137	0.0390	−0.0437	1.12

### Correlations between electron density in BCPs and interatomic O⋯Hal distances in trimethylamine-*N*-oxide complexes with halogens

**Fig. 4:**
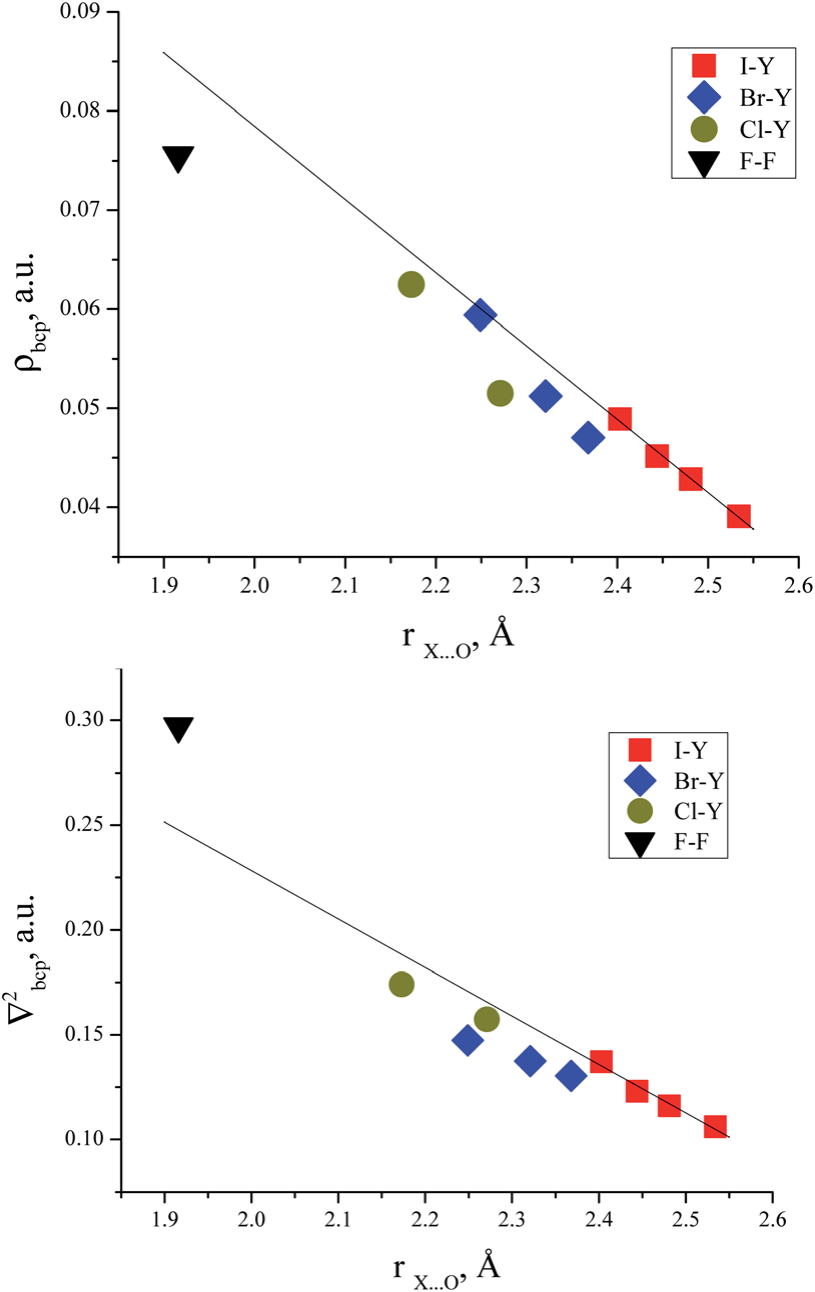
Electron density in BCP of O⋯X *vs.* interatomic distance of O⋯X (top) and Laplacian of electron density in BCP of O⋯X *vs.* interatomic distance (bottom). Linear approximations were made for the iodine-centred electrophiles.

There are some correlations between the electron density characteristics (*ρ*(*r*), ∇^2^*ρ*(*r*)) in the bond critical point for the oxygen–halogen short contact and interatomic distance “oxygen–halogen”. Good fit linear correlations are achievable only in separate groups of iodine- and bromine-centred electrophiles, though the general tendency is obvious for all the halogens heavier than fluorine (Fig. 4).

### Correlations between energies of complex formation and electron density characteristics in O⋯Hal bond critical points

**Fig. 5:**
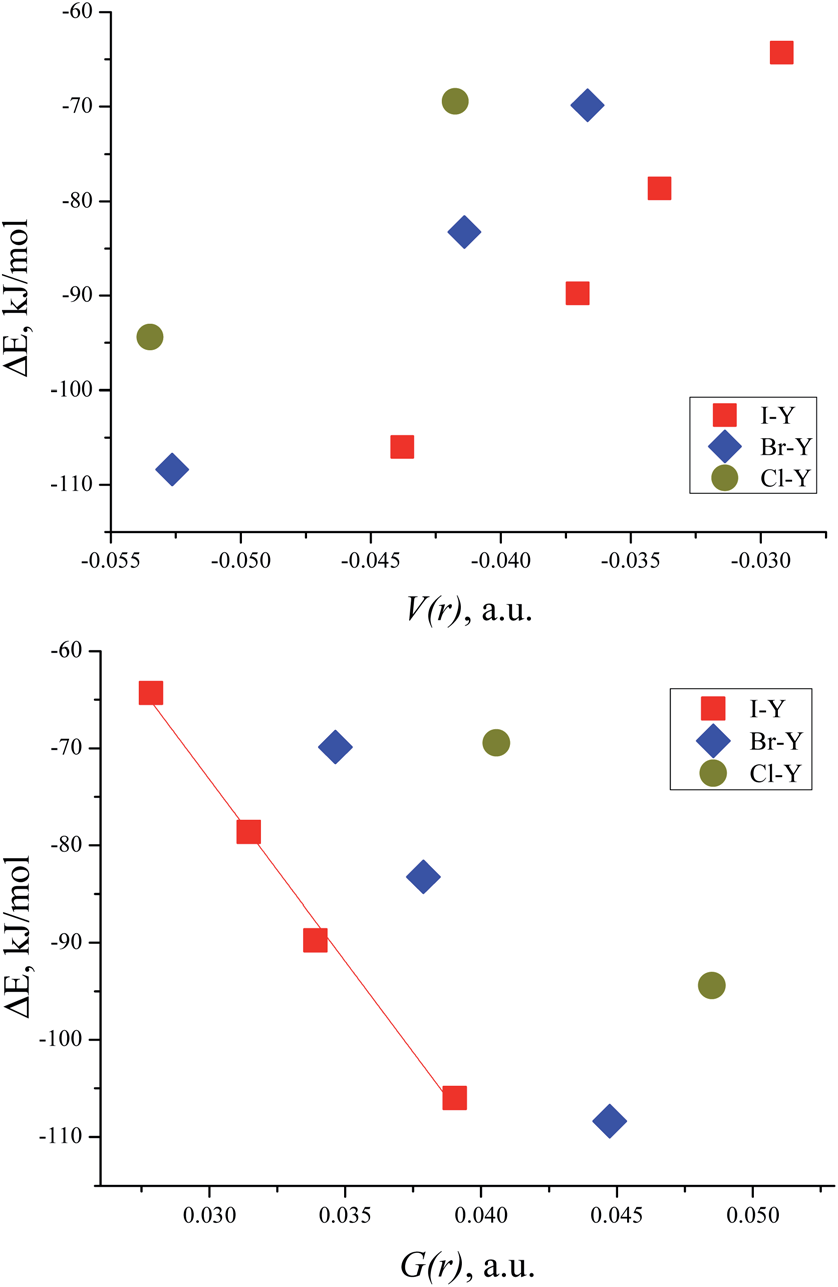
Interaction energy Δ*E* (BSSE corrected) for TMAO⋯X–Y *vs.* potential energy density *V*(*r*) in the BCP of the X⋯O bond (top) and *vs.* kinetic energy density *G*(*r*) in the BCP of the X⋯O bond (bottom).

The correlation between the energy of non-covalent interaction and electron density *ρ*(*r*) in the bond critical point was first highlighted in the case of hydrogen bonding^112^ and confirmed later many times.^113^ As other metrics of electron density in BCP (Hessian components, Laplacian, and densities of potential and kinetic energy) are defined by the value of *ρ*(*r*), and analogous correlations are also possible for them.^114^ For the TMAO⋯X–Y complexes under study, all dependencies of the interaction energies on the potential energy density *V*(*r*) in BCP are distinctly split into separate correlations for the iodine-, bromine- and chlorine-centred electrophiles (Fig. 5).

In the literature, it is very popular to compare the energetic metrics of halogen and hydrogen bonds. For the hydrogen bond, the very simple equation was proposed linking the potential energy density in the BCP and interaction energy^114^ as follows:
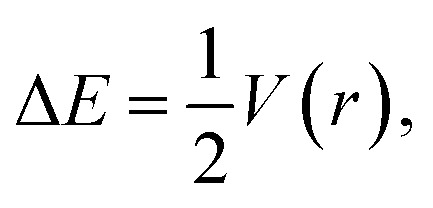
where the potential energy density is expressed in atomic units (a.u., hartree bohr^−3^), and the coefficients have dimensions of bohr^3^. Later, analogous equations were proposed for the kinetic energy density^115^ and Hessian components of the bonding contact BCP, and these equations were refined repeatedly for both hydrogen and halogen bonds. The scale values of 0.37 for the potential energy density and 0.448 for the kinetic energy density were proposed for hydrogen bonding.^115^ For the halogen bond, the corresponding values are 0.68 and 0.67, respectively.^65^ For the TMAO⋯X–Y complexes investigated herein, the dependence of the calculated interaction energies on the electron energy density in the X⋯O bond BCP was approximated with the linear equation:Δ*E* = *a* + *b* × *X*where *X* = *V*(*r*) or *G*(*r*). Scale values *a* and *b* for this equation for the electron energy densities in atomic units (a.u.) are presented in Table 10.

**Table 10:** Scale values of linear equations linking TMAO⋯X–Y interaction energies and electron energy densities in the X⋯O bond critical points

Complex	*a*/*V*(*r*), hartree	*b*/*V*(*r*), bohr^3^	*a*/*G*(*r*), hartree	*b*/*G*(*r*), bohr^3^
TMAO⋯Cl–Y	0.0073	0.809	0.022	−1.20
TMAO⋯Br–Y	0.006	0.906	0.023	−1.44
TMAO⋯I–Y	0.007	1.092	0.015	−1.43

Slope *b* (in bohr^3^) of these dependences on *V*(*r*) and *G*(*r*) for the halogen bonds in the TMAO⋯X–Y complexes significantly exceeds not only that typical for the hydrogen bond,^115^ but that proposed for molecular iodine also.^65^ Different correlations for different halogen centres were noted earlier.^116–118^ Obviously, any attempts to construct joint correlation “interaction energy–energy density in BCP” for hydrogen and halogen bonds are unlikely to be successful.

**Fig. 6:**
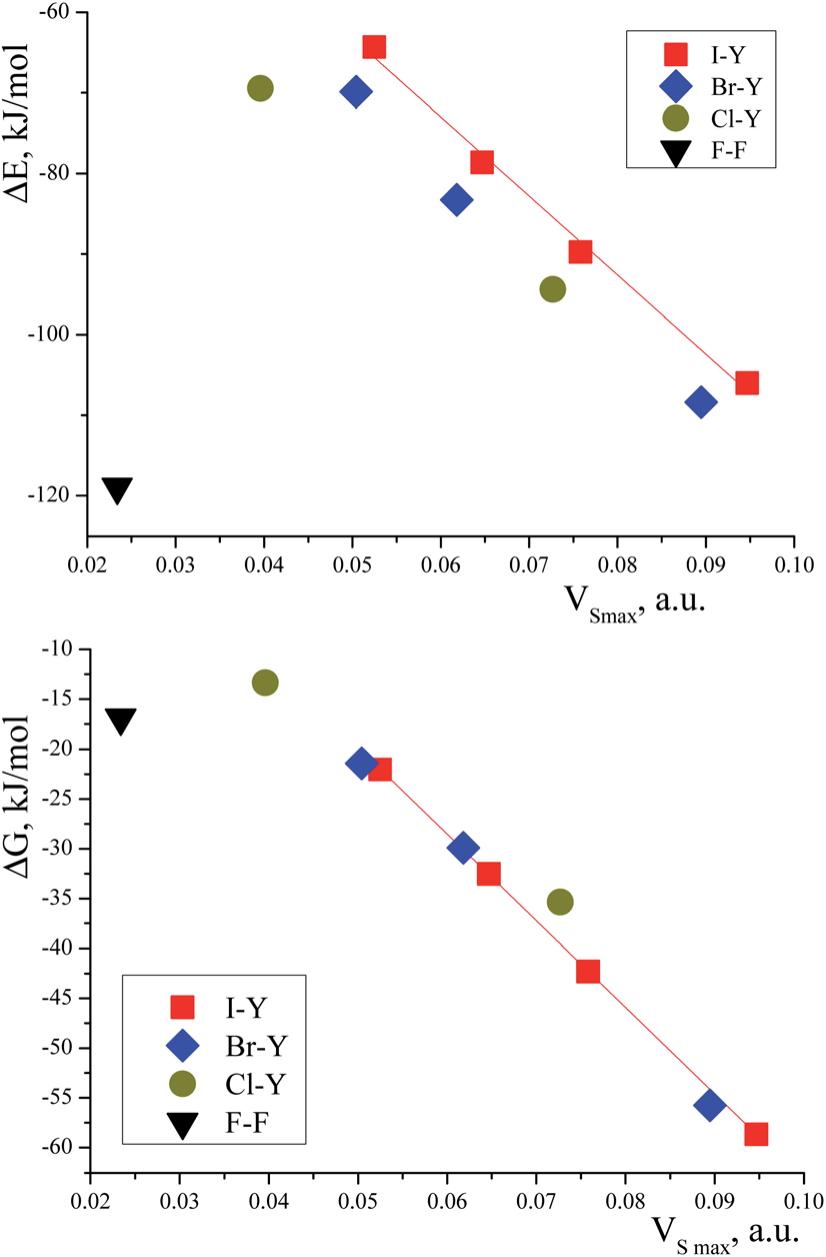
Complex TMAO⋯X–Y formation energy Δ*E* (BSSE corrected) *vs.* halogen X–Y maximum surface electrostatic potential *V*_S,max_ (top) and Gibbs energy *vs.* halogen X–Y maximum surface electrostatic potential *V*_S,max_ (bottom).

The correlation between the TMAO⋯X–Y interaction energies and maximal values of the halogen ESP (Table 7) is close to linear (Fig. 6, top). Within separate subgroups (iodine-centred and bromine-centred complexes) the linear fit is excellent.

The linear correlation between the interaction energy, Δ*E*, and surface potential distinctly indicates the main role of electrostatic interactions in the formation of the TMAO⋯X–Y complex. The contribution of these interactions to halogen bonding was extensively discussed in the literature.^92,97,108^ In the plot of Δ*E vs. V*_S,max_ (Fig. 6), the points for the fluorine, chlorine- and bromine-centred electrophiles lie under the linear fit for the iodine-centred electrophiles (at comparable ESP values, the interaction energy is more negative). Possibly, the C–H⋯X hydrogen bonds make their own contribution to the overall interaction of *N*-oxide and the halogen molecule. These hydrogen bonds were revealed with some uncertainties for the fluorine-, chlorine- and bromine-centred electrophiles, but not for the iodine-centred electrophiles (see later). This is very similar to multiple hydrogen bonds. In some visually simple structures (for example, chloroform–formaldehyde complex^113^), more than one non-covalent interaction was revealed, and good correlations were found between the interaction energy, Δ*E*, and the sum of electron density in the bond critical points,^113^ where the correlation worsened if one interaction was neglected.^113^ Oppositely, good correlations between the interaction enthalpy and negative logarithm of the association constants were found for *N*-halogenosaccharine–pyridine-*N*-oxide complexes bound with a halogen bond only, and in this case, structures with additional hydrogen bonds worsened the correlations.^90^

The correlation between the Gibbs energy, Δ*G*, and ESP looks shared for all the heavy halogens (Fig. 6, bottom). Perhaps, the energy contributions from the subtle C–H⋯X hydrogen bonds vanished at elevated temperature.

The data for the complex with difluorine could not be arranged in any correlation (as was often the case^119^). No correlations between the interaction energies and charge transfer were found, contrary to usual expectations. Earlier, good correlations were found between Δ*E* and charge transfer for several iodine complexes with pyridine-*N*-oxides,^91^ but this may have resulted from the perfect homogeneity of the dataset, with only diiodine being chosen as the halogen bond donor. In the case of a diverse set of halogen donors and heteroatom acceptors, correlations with charge transfer were absent.^120^

### Hydrogen bonds in TMAO complexes with chlorine and bromine

Bond critical points have been found in the structures of chlorine and bromine complexes for shortened C–H_β_⋯Cl and C–H_β_⋯Br contacts. In these BCPs, the electron density is very low; accordingly, the energy density is also low. The sum of distances between the BCP and attractors (hydrogen and halogen) exceeds interatomic distance between these atoms. Geometrically, the C–H_β_⋯X (X = Cl, Br) BCPs are close to the correspondent ring critical points (RCP 3, +1). This type of instability is not unusual for subtle hydrogen bonding accompanied by ring closure with a halogen bond^121^ or another hydrogen bond.^113^ In any case, these features of the BCPs make them uncertain and dictate the need for additional arguments.

**Fig. 7:**
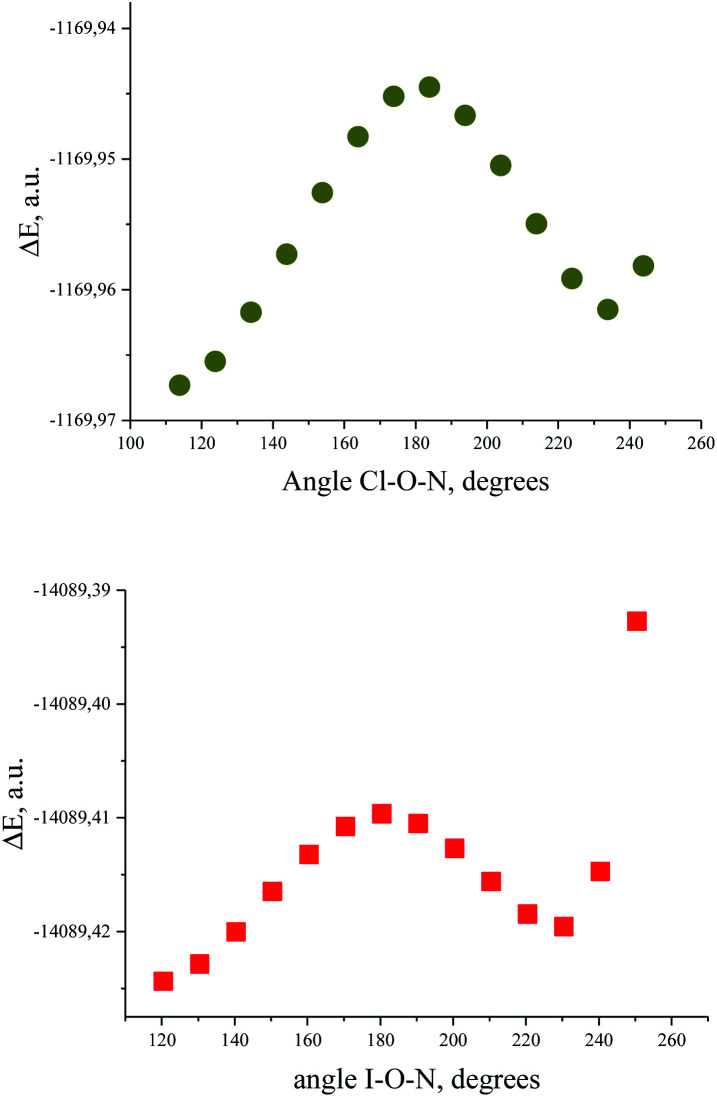
Full energy changes under enlargement of X–O–N angle for the TMAO⋯Cl–Cl complex (top) and for the TMAO⋯I–I complex (bottom).

**Fig. 8:**
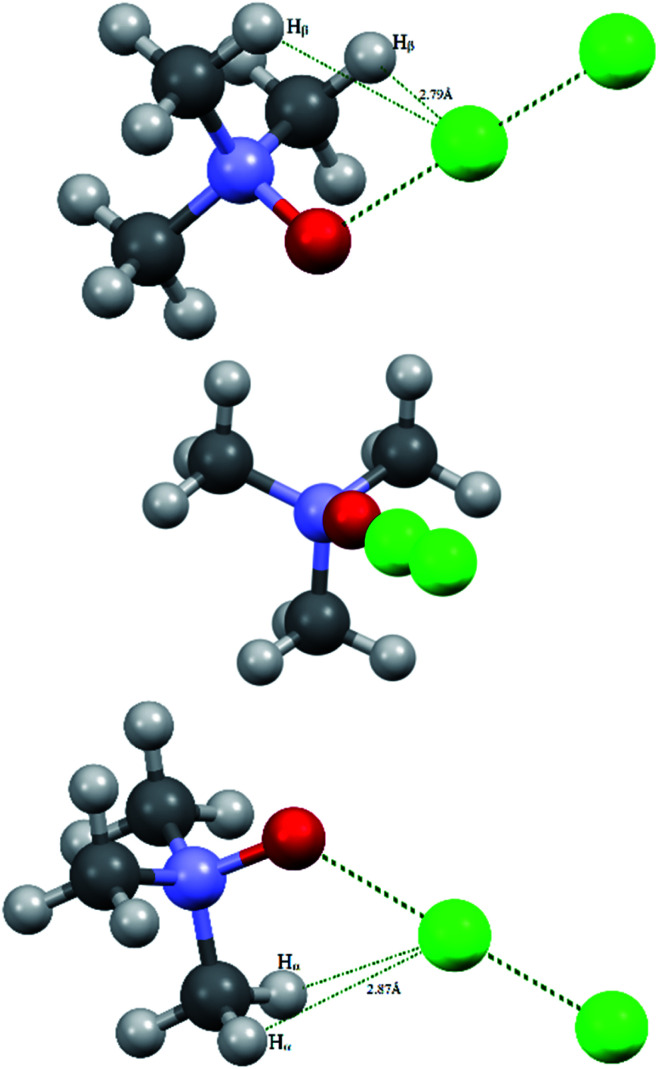
Structural evolutions of TMAO⋯Cl–Cl under forced enlargement of the Cl–O–N angle: global minimum Cl2-globmin (top); maximum Cl2-Max (centre); and local minimum Cl2-locmin (bottom).

The energy profile of the forced conformational changes carried out on the equilibrium TMAO⋯X–Y structures indirectly confirms the bonding character of the C–H_β_⋯X interactions (Fig. 7). In the global minimum of TMAO⋯Cl–Cl (equilibrium structure Cl2-globmin, Fig. 8, top) three atoms, Cl, O, and N, form a plane, and the H_α_-atom lies in the same plane, and the angle Cl–O–N is close to tetrahedral. Enlarging this angle by turning the Cl–Cl fragment in this plane, avoiding any other shifts in geometry, upon increasing the Cl–O–N angle (Fig. 8), two C_β_–H_β_⋯ Cl short contacts (2.79 Å) lengthen and finally break, and the full energy of the system becomes less negative. In the local maximum (Fig. 8, structure Cl2-Max, at the centre) four atoms, Cl–Cl⋯O–N, lie on the same straight line. Two longer contacts (2.87 Å) C_α_–H_α_⋯Cl appear with a further turn (Fig. 8 bottom, structure Cl2-locmin).

This procedure has to be considered as inversion of the Cl–O–N–C_α_ fragment from an anti-periplanar to sin-periplanar configuration. The new Cl2-locmin configuration is the minimum also, but local. The full system energy in this configuration is less negative compared to the global minimum with two hydrogen bonds from two methyl groups.

The TMAO⋯I–I complex behaves in the same way (Fig. 7, bottom). The energy difference for these two minima, global and local, was estimated to be 3.63 kcal mol^−1^ for TMAO⋯Cl–Cl and 3.01 kcal mol^−1^ for TMAO⋯I–I.

**Fig. 9:**
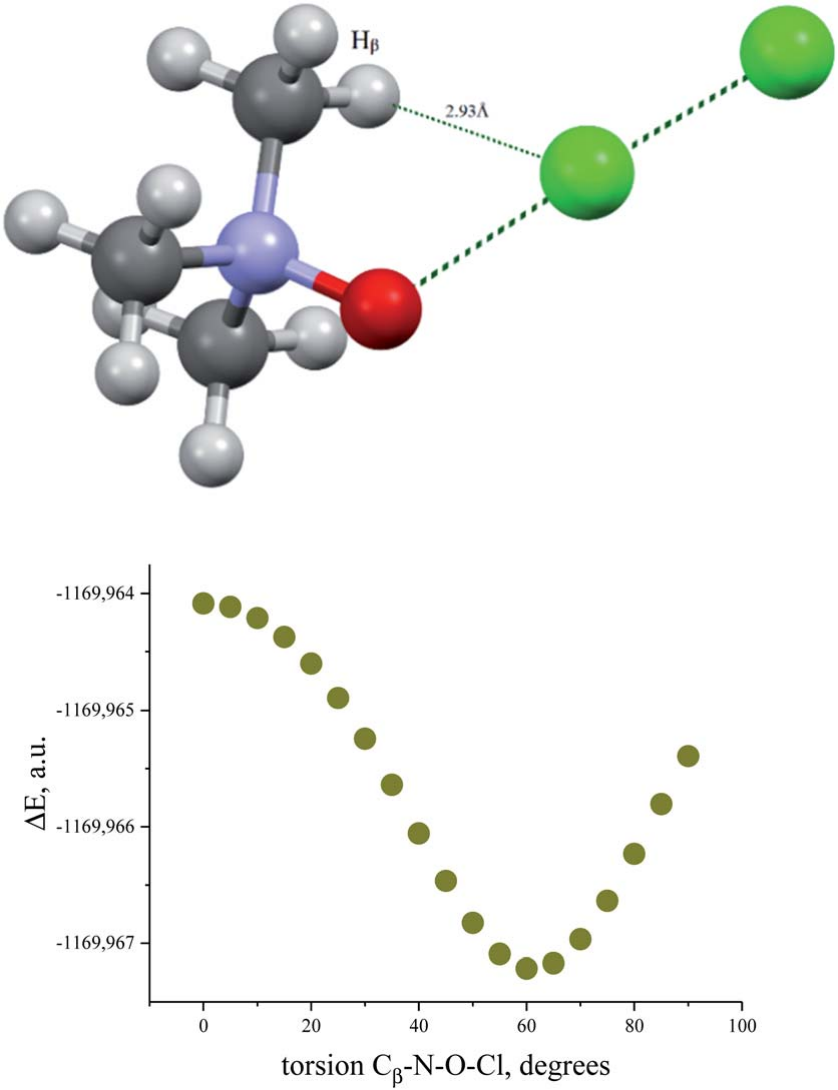
Structure of TMAO⋯Cl–Cl complex in the staggered conformation resulting after 60° degree turn around the N–O bond from the global minimum (top) and energy profile for the Cl–Cl fragment “slipping” around the N–O bond continuation (bottom).

Similar energetic changes were achieved by turning the chlorine molecule around the N–O bond (more precisely, around the line-continued N–O bond). Forced turning of the Cl–Cl fragment around the imaginary continuation of the N–O bond is equivalent to “slipping” of the Cl⋯O contact on the most negatively charged area on the surface of the oxygen atom. The energy profile for this movement is shown in Fig. 9, where the torsion ∠∠Cl–O–N–C_β_ = 60° coincides with the global minimum configuration. Any change in this torsion makes the whole structure less preferable energetically (Fig. 9, bottom). At the dihedral angle ∠∠Cl–O–N–C_β_ = 0°, the system accepts a staggered conformation, where the carbon and chlorine atoms are both sin-periplanar in relation to the N–O bond (Fig. 9, top).

The results of these two procedures (inversion and turn of the dichlorine fragment) demonstrate the significance of the role of the C–H⋯Cl hydrogen bond in the whole stability of the halogen-bonded complex. We did not find the C–H⋯I bond critical points for the hydrogen–iodine shortened contacts, although the experimental structure of the TMAO⋯ICl adduct unequivocally suggests the presence of this bonding. For iodine compounds, this is not unique. In the thoroughly studied experimental structure of the bis-pyridyl-*N*,*N*-dioxide complex with 1,4-diiodotetrafluorobenzene, the *ortho*-hydrogens of the pyridine ring are in close proximity to the iodine atoms, but the BCPs were not revealed.^89^ It is quite possible that these interactions are binding, and in some cases, it was supported with computations, but at the much higher MP2 level,^42^ or at the B3LYP-D3/def2-TZVP level.^121^

Obviously, fully correct investigation of these interactions with computations requires extended basis sets augmented with diffuse functions.^122^ At the current stage, we prefer to certify the fact of C–H⋯X bonding interactions in the structures of the TMAO⋯X–Y complexes and not to go beyond this.

**Fig. 10:**
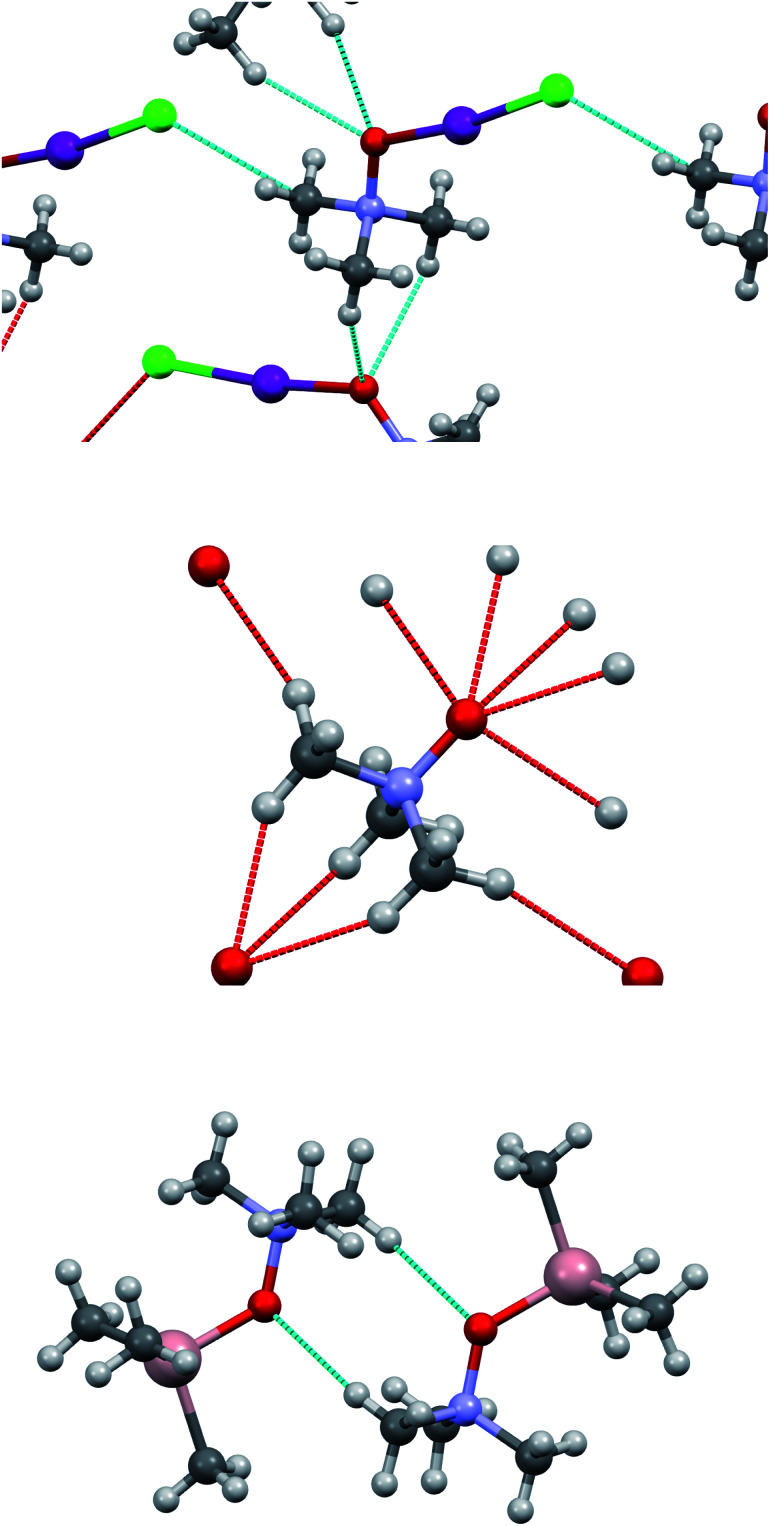
Short contacts in the crystal structure of adduct 3a (top), in TMAO (mid), and in trimethylamine-*N*-oxide–trimethylaluminum adduct (bottom).

The computed (and experimentally found) structures of the TMAO⋯X–Y complexes are not optimal for C–H…halogen bond formation, where the C–H⋯X angle is slightly less than 110° in comparison to the more frequent 150–170°. Nevertheless, such small C–H⋯X angles were identified (rarely) based on a statistical analysis provided for a huge number of real structures deposited in the Cambridge Structural Database.^86–88^ The analysis of the crystal structure revealed a C–H⋯O close contact network within the trimethylamine-*N*-oxide nucleophile sublattice and I–Cl⋯C_α_ shortened contacts (tetrel bond^123^) between TMAO⋯I–Cl units (Fig. 10). Information about these contacts is presented in Table 11.

**Table 11:** Interatomic distances and angles for short contacts in the crystals of TMAO⋯ICl adduct 3a

Contact	*r*_X⋯Y_, Å	*Σ*_VdW_, Å	(*Σ*_VdW_–*r*_X⋯Y_), Å	∠C–H–X, degrees
C–H⋯O	2.602	2.72	0.118	152.2
C–H⋯Br	3.071	3.18	0.109	108.5
I–Cl…C_α_	3.420	3.45	0.030	135.16 (∠I–Cl–C_α_); 157.74 (∠Cl–C_α_–N)

The contacts C–H⋯O in adduct 3a are longer than in the initial trimethylamine-*N*-oxide^61^ (the shortest H⋯O distance 2.458 Å), and the whole set is less intricate compared with TMAO itself. Accordingly, the structure of 3a resembles a trimethylamine-*N*-oxide–trimethylaluminum adduct^124^ with only one C–H⋯O contact per molecule and H⋯O distance of 2.685 Å, or TMAO–dihydrate with H⋯O distance of 2.661 Å.^125^ Seemingly, the C–H⋯O interactions affect the relative positions of TMAO in the nucleophile sublattice but do not seem to be as significant for the TMAO–halogen interactions as halogen bonding (oxygen⋯iodine).

## Summary

In conclusion, in the study of the dihalogen–trimethylamine-*N*-oxide interactions, molecular complexes 3a and 3b were found to be stable under ambient conditions and have a 1 : 1 stoichiometry of the nucleophile to interhalogens ICl and IBr, respectively. Oxygen-centred nucleophiles are not prone to produce ionic complexes with halogens in the solid phase spontaneously (in contrast to nitrogen-, sulphur- and other heteroatom-centred nucleophiles). Thus, other tactics must be chosen for imaginable ionic structures. From computation, it is clear, that (1) the B3LYP/DGDZVP computations adequately reproduce the geometry and electron structure of trimethylamine-*N*-oxide and diatomic halogens and interhalogens; (2) the B3LYP/DGDZVP computations plausibly represent the geometry and electron structure of the trimethylamine-*N*-oxide complexes with diatomic halogens and interhalogens; and (3) the electrostatically driven halogen bond with oxygen is the main driving force for the formation of the TMAO⋯X–Y complex, assisted by the C–H⋯X hydrogen bond. Generally, (1) trimethylamine-*N*-oxide complexes with diatomic halogens and interhalogens are the strongest between known complexes with “oxygen⋯halogen” non-covalent bonds; (2) the trimethylamine-*N*-oxide affinity to halogens is comparable with its affinity to hydrogen bond donors; (3) it is highly probable that other trialkylamine-*N*-oxides possess high affinity to halogens; and (4) this high affinity to halogens (and other halogen bond donors) may be of high importance for biologic systems and organs enriched with both trimethylamine-*N*-oxide and halogen species (seawater organisms, gastrointestinal tract, and liver).

## Conflicts of interest

There are no conflicts to declare.

## Supplementary Material

RA-011-D0RA08165E-s001
